# Percutaneous Endoscopic Interlaminar Discectomy *via* Laminoplasty Technique for L_5_–S_1_ Lumbar Disc Herniation with a Narrow Interlaminar Window

**DOI:** 10.1111/os.12978

**Published:** 2021-03-16

**Authors:** Tian‐long Wu, Jing‐hong Yuan, Jing‐yu Jia, Ding‐wen He, Xin‐xin Miao, Jian‐jian Deng, Xi‐gao Cheng

**Affiliations:** ^1^ Department of Orthopaedics The Second Affiliated Hospital of Nanchang University Nanchang China; ^2^ Institute of Orthopaedics of Jiangxi Province Nanchang China; ^3^ Institute of Minimally Invasive Orthopaedics of Nanchang University Nanchang China

**Keywords:** Discectomy, Percutaneous, Endoscopy, Osteoarthritis, Spine

## Abstract

**Objective:**

To improve the treatment effect of patients with L_5_S_1_ lumber disc herniation (LDH) with a narrow interlaminar window, we proposed an alternative approach to percutaneous endoscopic interlaminar discectomy (PEID) *via* the laminoplasty technique.

**Methods:**

Fifteen L_5_S_1_ LDH patients (7 men and 8 women; age range, 22 to 56 years; median age, 34 years; 9 left, 6 right) were enrolled in the present study retrospectively. The interlaminar windows of all patients were narrow (the transverse diameter of the L_5_S_1_ interlaminar window is equal to or less than that of L_4–5_). Percutaneous laminoplasty and endoscopic interlaminar discectomy surgery were undergone by all patients from July 2018 to July 2019. All operations were completed under local anesthesia. The target laminoplasty area was the safety zone, use of which avoids both transverse and exit nerve roots. Under fluoroscopic guidance or clear endoscopic visualization, the trephines were used to enlarge the interlaminar window, which allowed the working cannula to enter the spinal canal but avoid nerve roots and the dural sac. The preoperative/postoperative visual analogue scale (VAS) scores and Oswestry disability index (ODI) were statistically analyzed. The modified MacNab criterion was used to assess the clinical effects. The radiological outcomes were evaluated by MRI and CT. SPSS 19.0 software was used for the statistical evaluation.

**Results:**

The operative time ranged from 70 to 120 min, with a median time of 92 min, and the fluoroscopy times ranged from 8 to 12, with a median of 9.7 times. The body mass index (BMI) of patients ranged from 18.10 to 26.06, with a median of 22.04. All patients were followed up in the outpatient department for at least 12 months after surgery. At the last follow up, the average VAS‐Back score of the study patients was reduced from 5.33 ± 2.09 to 2.00 ± 1.20 (*P* < 0.001) and the average VAS‐Leg score was reduced from 7.53 ± 1.69 to 1.47 ± 0.92 (*P* < 0.001). The average ODI scores improved from 47.87 ± 11.41 to 12.93 ± 3.24 (*P* < 0.01). According to the modified MacNab criteria, 11 cases achieved excellent results and 4 cases achieved good results. All of the operations were successful. There wertr no nerve root injuries, dural tears, or other complications.

**Conclusion:**

The laminoplasty approach for PEID provides a safe and useful alternative for the treatment of L5–S1 LDH patients with a narrow interlaminar window.

## Graphical abstract

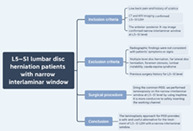

The authors used an innovative method to more safely and effectively perform PIED on LDH patients with L5–S1 with a narrow interlaminar window.

## Introduction

Lumbar disc herniation (LDH) is a common cause of lower back and leg pain, which results in medical and economic burdens for families and society. Due to the modern sedentary lifestyle, the incidence of LDH is increasing. When expectant treatment fails, surgical treatment is often required, which may by open surgery or minimally invasive surgery. Open lumbar discectomy often leads to iatrogenic damage of the facet joints and paraspinal structures, causing muscle denervation and atrophy, segmental instability, and long‐term lumbodorsal muscular pain postoperatively. These factors seriously affect the clinical curative effect and patient satisfaction.

With the rapid development of minimally invasive spine surgery, the technique of spinal endoscopy has made revolutionary progress, especially for percutaneous endoscopic lumbar discectomy (PELD). The application of PELD has many advantages, including reduced paraspinal muscle trauma, minimal postoperative instability, lower blood loss, smaller surgical wound, and faster recovery^1^. Percutaneous endoscopic therapy for disc herniation can be divided into the transforaminal approach^2^ (percutaneous endoscopic transforaminal discectomy [PETD]) and the interlaminar approach^3^ (percutaneous endoscopic interlaminar discectomy [PEID]). For L_5_S_1_ level, PEID, which can escape the blockade of crista iliaca, has advantages over PETD, including reducing the difficulty of puncture, a faster puncture orientation, a shorter operation time, and less intraoperative radiation exposure^4^, ^5^, ^6^, ^7^.

**Fig. 1:**
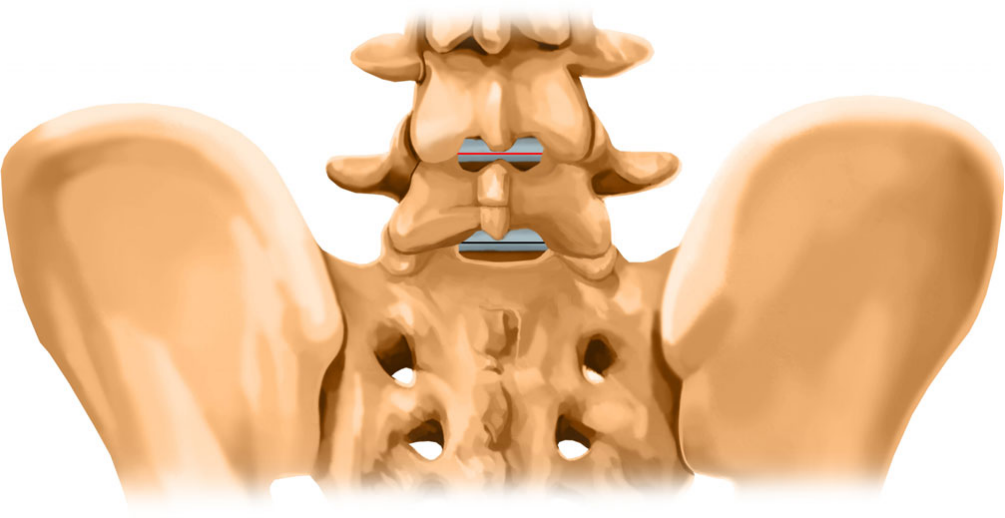
Narrow interlaminar window of L_5_S_1_ level: The red line represents the transverse diameter of the L_4–5_ interlaminar window, the black line represents the transverse diameter of the L_5_S_1_ interlaminar window. Generally, the black line is longer than the red line. When the black line is equal to or less than the red line, we define it as a narrow interlaminar window of L_5_S_1_ level.

Currently, PEID is usually performed in the L_5_S_1_ segment because the interlaminar window is the widest^8^. However, some patients have a narrow interlaminar window at L_5_S_1_ level (Fig. 1). For L_5_S_1_ segments with a narrow interlaminar window, PEID might not be able an option because of the risk of dural tear^9^ and nerve root injury^10^. To provide a safe and practicable option for L_5_S_1_ LDH cases with a narrow interlaminar window, we propose full‐endoscopic interlaminar discectomy *via* the laminoplasty technique, which can effectively and safely allow the working cannula to enter the spinal canal but avoid the nerve roots and dural sac.

In this study, the aims were: (i) to introduce PEID *via* a novel laminoplasty technique for the treatment of L_5_S_1_ LDH patients with a narrow interlaminar window; (ii) to evaluate the feasibility of this new approach; and (iii) to determine the safety and effectiveness of this new strategy.

## Materials and Methods

### Inclusion and Exclusion Criteria and Patients

The inclusion criteria were as follows: (i) low back pain and history of sciatica; (ii) L_5_S_1_ LDH confirmed by CT and MRI; (iii) the anterior–posterior X‐ray image confirmed the narrow interlaminar window at L_5_S_1_ level; and (iv) standard conservative treatment for at least 3 months had failed to relieve recurrent pain.

The exclusion criteria were as follows: (i) radiographic findings were not consistent with patients’ symptoms or signs; (ii) multiple level disc herniation, far lateral disc herniation, foramen stenosis, lumbar instability, or cauda equina syndrome; (iii) previous surgery history for L_5_S_1_ level; (iv) the anterior–posterior X‐ray image determined patients who were able to accept conventional PELD for L_5_S_1_ level.

Based on the inclusion and exclusion criteria, 15 L_5_S_1_ LDH patients (7 men and 8 women; age range, 22 to 56 years; median age, 34 years) with a narrow interlaminar window who accepted our novel approach at The Second Affiliated Hospital of Nanchang University from July 2018 to July 2019 were included in the present study. Written informed consent was obtained from all patients. The study was approved by the Ethics Committee of the Second Affiliated Hospital of Nanchang University.

### Surgical Procedures

#### Step 1. Anesthesia and Position

Patients were placed in the prone decubitus position on a radiolucent operating table. Local anesthesia and intravenous sedation were chosen for this strategy. The patients communicated with the surgeon throughout the procedure.

#### Step 2. Approach

**Fig. 2:**
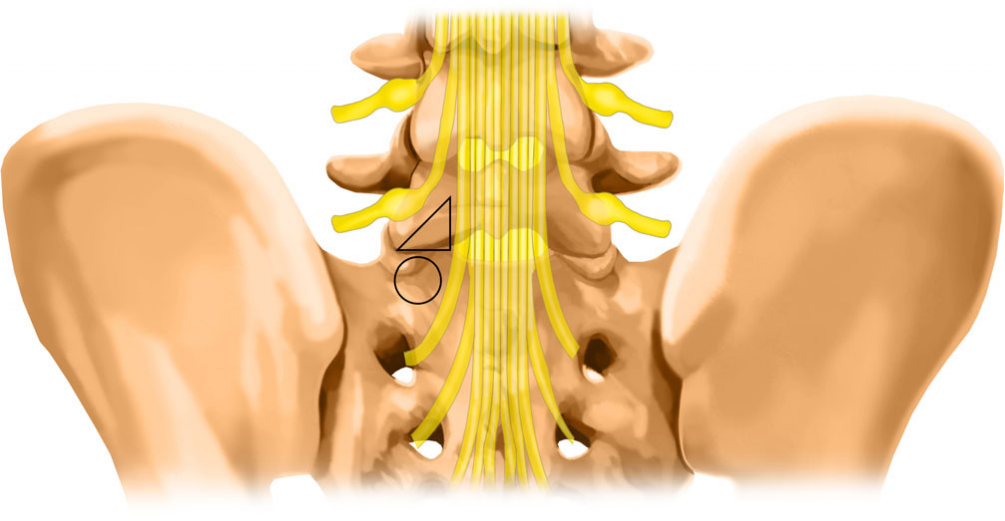
Safety zone: The triangle (the lamina above the pedicle) represents the safety zone and the circle represents the S1 pedicle. Using the safety zone avoids both transverse and exit nerve roots.

**Fig. 3:**
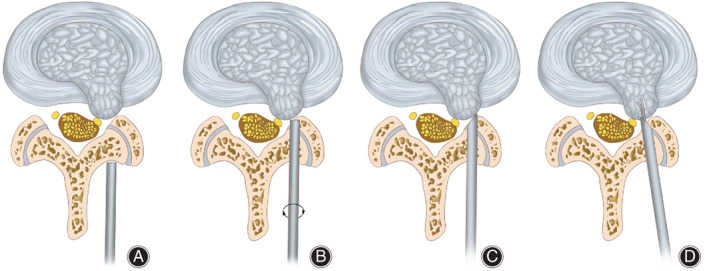
Surgical diagrams. (A) The trephine was located in the safety zone. (B) The trephine was advanced with careful rotation for laminoplasty. (C) The working cannula reached the herniated disc tissue *via* the spinal canal. (D) By maneuvering the working cannula, neural tissues were protected and the herniated disc was removed.

The skin was marked under fluoroscopic guidance. The target point was the safety zone (Fig. 2). The skin entry point was the midpoint of the interlaminar window. The connection line of the target point and the entry point was the direction of the puncture. Following the designed puncture trajectory, a puncture needle was inserted, and skin incision and soft tissue dilatation were performed. A trephine was located in the safety zone (Fig. 3A).

#### Step 3. Laminoplasty

Laminoplasty was performed using the trephine to enlarge the interlaminar window. The trephine should be advanced with careful rotation under fluoroscopic guidance (Fig. 3B) or clear endoscopic visualization. The bone of the lamina was cut off and could be taken out along with the trephine. Laminoplasty could be repeated if necessary.

#### Step 4. Channel Placement

The working channel was then introduced (Fig. 3C), and the endoscope was inserted through cannula. The remaining lamina and ligamentum flavum could be easily removed under clear endoscopic visualization. The working cannula and endoscope were located at the shoulder of the nerve root.

#### Step 5. Decompression

After confirmation of the structures under clear endoscopic visualization, the herniated disc was removed using various graspers (Fig. 3D). The neural tissues were pushed and, thus, protected by maneuvering the working cannula, which allowed the fragments in the ventral and axillary part of the nerve root to be removed. Plasma radiofrequency was used to stop bleeding and ablation of the disc. The operation was completed when the nerve root had been explored and released.

### Data Collection

The operation time and fluoroscopy times were documented. Complications during and after the operation were recorded to evaluate the safety of the surgery. The 10‐point visual analogue scale (VAS) was adopted to assess back pain (VAS‐Back) and leg pain (VAS‐Leg) at the following time points: preoperation, 1st postoperative day, 3rd postoperative month, and 12th postoperative month. The Oswestry disability index (ODI) was used to evaluate patient functional status at the following time points: preoperation and at the 12th postoperative month. The modified MacNab criteria at the 12th postoperative month was recorded to evaluate the early clinical efficacy. Assessments were performed by an independent observer.

### Subgroup Analysis

In this study, subgroup analysis was applied to evaluate the effectiveness of the PEID *via* laminoplasty according to age, gender, BMI, left/right intervertebral disc herniation, and the length of the operation.

### Clinical Assessment

#### Visual analogue scale

The VAS score system has been widely used in recent research to assess lower back pain. The VAS score system (score from 0 to 10) is calculated as: 0 means painless; 1–3 indicates mild pain that the patient can tolerate; 4–6 indicates that the patient is in pain that could be tolerated and is able to sleep; and 7–10 indicates that the patient has severe pain and is unable to endure the pain.

#### Oswestry Disability Index

The ODI has been widely used to assess patients’ disability as a result of lower back pain. The ODI score system includes 10 sections: pain intensity, personal care, lifting, walking, sitting, standing, sleeping, sex life, social life, and traveling. For each section of six statements, the total score is 5. Intervening statements are scored according to rank. If more than one box is marked in each section, the highest score is taken. If all 10 sections are completed, the score is calculated as follows: total scored out of total possible score × 100. If one section is missed (or not applicable) the score is calculated as: (total score/(5 × number of questions answered)) × 100%. Scores of 0%–20% are considered mild dysfunction, 21%–40% is moderate dysfunction, 41%–60% is severe dysfunction, and 61%–80% is considered a disability. For cases with scores of 81%–100%, patients are either long‐term bedridden or exaggerating the impact of pain on their life.

#### The modified MacNab Criteria

The modified MacNab criteria were used to evaluate the surgical outcomes: excellent means no pain and no restriction of movement, so that the patient can work normally; good means occasional pain, so that the patient can work normally; fair means slight progress; poor means no progression.

### Statistical Analysis

All data were statistically analyzed using SPSS software (Version 19.0; IBM). Continuous variables were presented as mean ± standard deviation. Student's *t*‐test was used to compare the continuous variables, such as VAS‐Back, VAS‐Leg, and ODI scores, between different time points. A positive significance level was assumed at a *P*‐value of less than 0.05.

## Results

### Surgical Information

The laminoplasties were successful completed and all patients received significant pain relief after the surgery. The operative time ranged from 70 to 120 min, with a median time of 92 min. The average fluoroscopy times was 9.7 times.

### Clinical Outcomes

All 15 patients were followed up for at least 12 months.

*VAS‐Back score*. The average VAS‐Back score was reduced from 5.33 ± 2.09 to 2.00 ± 1.20 (*P* < 0.05) at the last follow up.

*VAS‐Leg*. The average VAS‐Leg score was reduced from 7.53 ± 1.69 to 1.47 ± 0.92 (*P* < 0.05) at the last follow up.

*ODI scores*. As for functional improvement, the average ODI scores improved from 47.87 ± 11.41 to 12.93 ± 3.24 (*P* < 0.05) at the last follow‐up.

*MacNab criteria*. According to the modified MacNab criteria, 100% of patients (11 excellent and 4 good) had an excellent or good recovery and no poor result was reported.

*Complications*. With respect to complications, no nerve root injuries, dural tears, lamina fractures, infections, or intraspinal hematomas were observed.

### Subgroup Analysis

No recurrence was observed at follow‐up. No significant differences were found in the subgroup analysis (Table 1).

**TABLE 1:** Subgroup analysis

Subgroup	Number of patients (%)	VAS‐Back			VAS‐Leg			ODI		
		Preoperative	12 months	*P‐*Value	Preoperative	12 months	*P‐*value	Preoperative	12 months	*P*‐value
All patients	15 (100)	5.33 ± 2.09	2.00 ± 1.20	< 0.001	7.53 ± 1.69	1.47 ± 0.92	< 0.001	47.87 ± 11.41	12.93 ± 3.24	< 0.001
Gender										
Male	7 (46.67)	6.14 ± 2.19	2.00 ± 1.00	0.001	8.29 ± 1.11	1.71 ± 0.49	< 0.001	44.14 ± 11.10	12.29 ± 3.04	< 0.001
Female	8 (54.33)	4.63 ± 1.85	2.00 ± 1.41	0.009	6.88 ± 1.89	1.25 ± 1.17	< 0.001	51.13 ± 11.36	13.50 ± 3.51	< 0.001
Age										
Older	8 (54.33)	5.88 ± 2.42	2.13 ± 1.25	< 0.001	8.00 ± 1.31	1.50 ± 0.93	< 0.001	49.50 ± 11.19	12.25 ± 3.33	< 0.001
Young	7 (46.67)	4.71 ± 1.60	1.86 ± 1.22	0.023	7.00 ± 2.00	1.43 ± 0.98	< 0.001	46.00 ± 12.26	13.71 ± 3.20	< 0.001
BMI										
Higher	9 (60.00)	5.11 ± 2.42	2.00 ± 1.32	0.005	7.44 ± 1.94	1.67 ± 0.71	< 0.001	43.67 ± 10.82	11.78 ± 3.15	< 0.001
Lower	6 (40.00)	5.67 ± 1.63	2.00 ± 1.10	0.002	7.67 ± 1.37	1.17 ± 1.17	< 0.001	54.17 ± 9.91	14.67 ± 2.73	< 0.001
Right/Left										
Right	6 (40.00)	5.86 ± 2.41	2.71 ± 0.95	0.027	7.29 ± 1.98	1.43 ± 0.98	< 0.001	47.57 ± 13.35	13.14 ± 3.13	< 0.001
Left	9 (60.00)	4.88 ± 1.81	1.38 ± 1.06	< 0.001	7.75 ± 1.49	1.50 ± 0.93	< 0.001	48.13 ± 10.37	12.75 ± 3.54	< 0.001
Operative time										
Longer	8 (54.33)	5.75 ± 2.71	2.50 ± 1.31	0.01	7.88 ± 1.89	1.88 ± 0.83	< 0.001	46.38 ± 11.64	12.50 ± 3.42	< 0.001
Shorter	7 (46.67)	4.85 ± 1.07	1.43 ± 0.79	0.01	7.14 ± 1.46	1.00 ± 0.82	< 0.001	49.57 ± 11.82	13.43 ± 3.21	< 0.001

BMI, body mass index; ODI, Oswestry disability index; VAS, visual analogue scale.

### Typical Cases

**Fig. 4:**
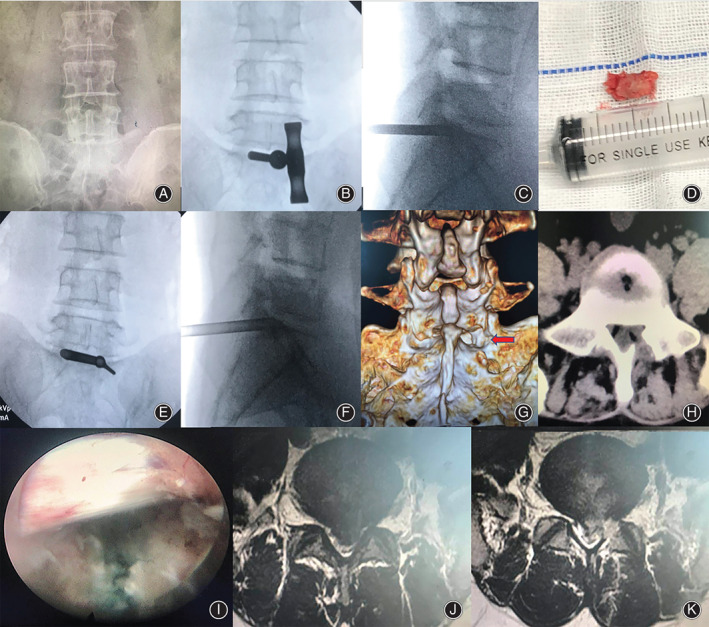
Typical case one. (A) Lumbar anterior–posterior X‐ray image. Narrow interlaminar window of L_5_S_1_ level. (B and C) The trephine was used for laminoplasty at the safety zone. (D) Resected bone from the safety zone. (E and F) Working cannula was inserted at the safety zone. (G and H) Postoperative CT scan revealed laminoplasty (arrow). (I) S_1_ nerve root was completely released. (J and K) Preoperative and postoperative MRI showed removal and good decompression of the S_1_ nerve root and dura.

*Case one*. A 36‐year‐old man was admitted to our department for severe left leg radicular pain of nearly 4 months. This patient was diagnosed with L_5_S_1_ LDH with a narrow interlaminar window. The laminoplasty was made and a herniated mass was completely removed during the PEID surgery. This patient received immediate pain relief and was discharged from hospital on the 3rd postoperative day. During the follow up, the functional improvement was satisfactory (Fig. 4).

**Fig. 5:**
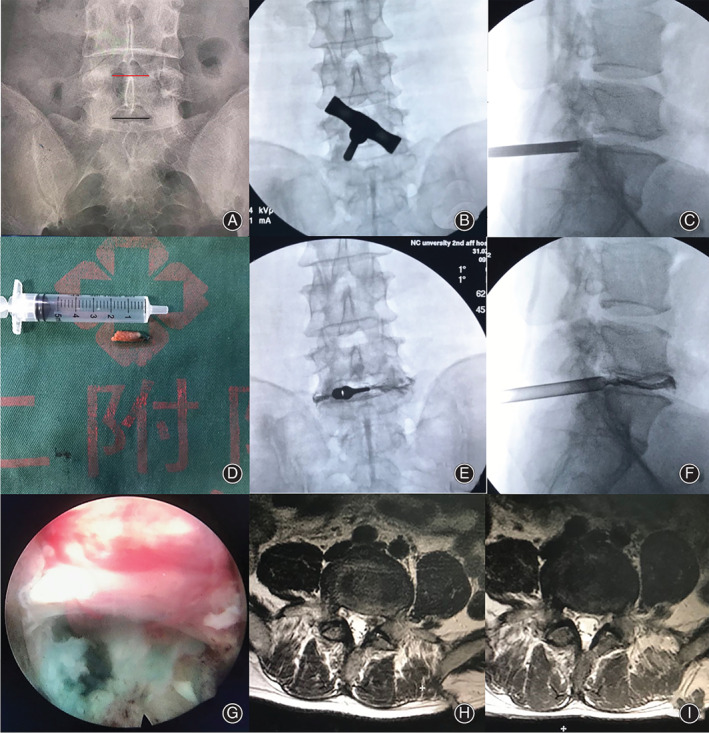
Typical case two. (A) Lumbar anterior–posterior X‐ray image. Narrow interlaminar window of L_5_S_1_ level. (B and C) The trephine was used for laminoplasty at the safety zone. (D) Resected bone from the safety zone. (E and F) Working cannula was inserted at the safety zone. (G) The S_1_ nerve root was completely released. (J and K) Preoperative and postoperative MRI showed good decompression of the left S_1_ nerve root.

*Case two*. A 44‐year‐old man complained of severe left leg pain for 3 months. MRI showed a herniated disc on the left side at L_5_S_1_ level. The interlaminar window was narrow according to X‐ray images. PEID was successfully performed after laminoplasty. He received immediate pain relief and was discharged from hospital on the 2nd postoperative day. During the follow‐up, the functional improvement was satisfactory (Fig. 5).

**Fig. 6:**
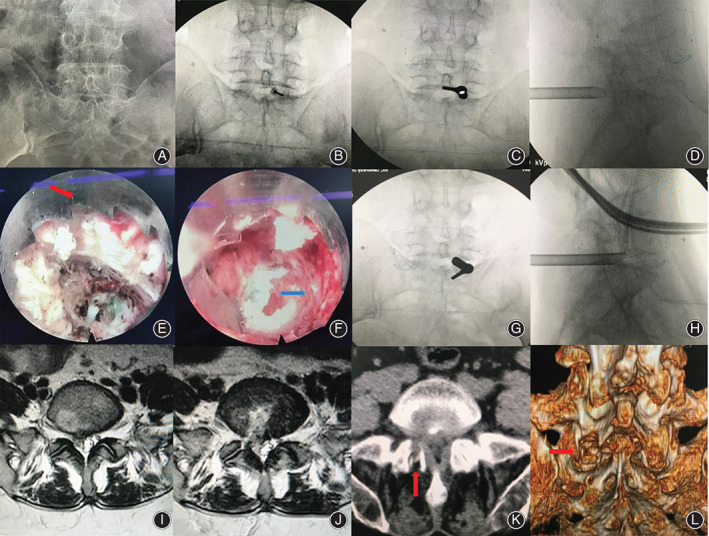
Typical case three. (A) Lumbar anterior–posterior X‐ray image. Narrow interlaminar window of L_5_–S_1_ level. (B, C, and D) The target puncture point and working cannula were located at the safety zone. (E and F) Endoscopic visible trephine (red arrow) was used for the laminoplasty (blue arrow). (G and H) Working cannula reached herniated disc tissue *via* the spinal canal under endoscopic observation. (I and J) Preoperative and postoperative MRI showed removal and good decompression of the S_1_ nerve root and dura. (K and L) Postoperative CT scan revealed laminoplasty (arrow).

*Case three*. A 56‐year‐old woman with severe right leg radicular pain for 6 months. L_5_S_1_ LDH with a narrow interlaminar window was diagnosed according to X‐ray and MRI images. The laminoplasty was performed with endoscopic visible trephines and a herniated mass was completely removed. She received immediate pain relief and was discharged from hospital on the 3rd postoperative day. During the follow‐up, the functional improvement was reported as satisfactory (Fig. 6).

## Discussion

Percutaneous endoscopic interlaminar discectomy has been widely used in the management of L_5_–S_1_ LDH, with the advantages including escaping the blockade of crista iliaca, a faster puncture orientation, decreased intraoperative blood loss, a shorter operation time, and less intraoperative radiation exposure^4^, ^5^, ^6^, ^7^. Normally, PEID can be performed at L_5_S_1_ level because the interlaminar window of L_5_S_1_ is the largest^8^. Once the transverse diameter of the L_5_S_1_ interlaminar window is equal to or less than that of L4–5, we define it as a narrow interlaminar window of L_5_S_1_. At present, there are two main techniques for PEID: having a working channel in the ligamentum flavum^1^ and having a working channel directly into the spinal canal^11^. For the L_5_–S_1_ LDH with a narrow interlaminar window, neither of these two techniques can be performed because of the blockade of the nerve root and the dural sac and damage can easily occur. Sencer *et al*.^12^ reported a 3.1% rate of nerve root injury and a 3.7% incidence of dural tear due to improper operation during the interlaminar approach. For patients with a narrow interlaminar window, the risk could be higher than reported. Therefore, achieving efficient interlaminar window enlargement while minimizing radiation exposure and protecting the nerves is important.

### Features of the Technique

In the current study, we introduced the PEID *via* a laminoplasty approach for L_5_S_1_ LDH with a narrow interlaminar window. Laminoplasty at the safety zone, use of which avoids both transverse and exit nerve roots, allowed us to efficiently enlarge the interlaminar window without the redundancy and complications associated with trephines. The trephine was advanced with careful rotation under fluoroscopic guidance and the patients communicated with the surgeon throughout the procedure. Our clinical results, with no severe complications occurring, confirmed the safety of the laminoplasty. With the application of an endoscopic high‐speed drill^13^ and endoscopic visible trephines, the surgeon could accomplish this laminoplasty under direct endoscopic observation, and the safety of laminoplasty could be further assured. Meanwhile, laminoplasty did not destroy facet joints; thus, the stability of the lumbar spine was not affected.

L_5_S_1_ disc herniations can be divided into three types: shoulder type, ventral type, and axilla type^14^. In this study, the working cannula and endoscope were located at the shoulder of the nerve root. By maneuvering the working cannula, the neural tissues were pushed and protected, which allowed the fragments in the ventral and axillary part of the nerve root to be removed. For the axillary type, the axillary side of the S_1_ nerve root needed to be explored.

### Functional Outcomes

All patients experienced significant pain reduction and functional improvement after surgery and all of the patients (100%) had obtained excellent or good recovery at the last follow up. Therefore, our study showed that PEID *via* a laminoplasty approach could manage a case of L_5_S_1_ LDH with a narrow interlaminar window efficiently; and, as a result, this technique might be a feasible alternative to treat L_5_S_1_ LDH with a narrow interlaminar window.

### Limitations

Some limitations should be considered when interpreting our data. The retrospective design might lead to selection bias and the small sample size might reduce the stringency of our results. In addition, there was no comparison with other surgical techniques in this study. Therefore, we should further validate this novel approach in clinical trials, including prospective and multiple‐center studies. However, it should be noted that we aimed to introduce a new endoscopic discectomy strategy for the treatment of L_5_S_1_ LDH with a narrow interlaminar window, and our results demonstrated that this strategy was effective and safe.

## Conclusion

The laminoplasty approach for PEID provides a safe and useful alternative for the treatment of L_5_S_1_ LDH with a narrow interlaminar window.

## References

[1] Ahn Y . Endoscopic spine discectomy: indications and outcomes. Int Orthop, 2019, 43: 909–916. 30612170 10.1007/s00264-018-04283-w

[2] Yeung AT , Tsou PM . Posterolateral endoscopic excision for lumbar disc herniation: surgical technique, outcome, and complications in 307 consecutive cases. Spine, 2002, 27: 722–731. 11923665 10.1097/00007632-200204010-00009

[3] Ruetten S , Komp M , Godolias G . A new full‐endoscopic technique for the interlaminar operation of lumbar disc herniations using 6‐mm endoscopes: prospective 2‐year results of 331 patients. Minim Invasive Neurosurg, 2006, 49: 80–87. 16708336 10.1055/s-2006-932172

[4] Nie H , Zeng J , Song Y , *et al*. Percutaneous endoscopic lumbar discectomy for L5–S1 disc herniation via an Interlaminar approach versus a Transforaminal approach: a prospective randomized controlled study with 2‐year follow up. Spine, 2016, 41: B30–B37. 27454540 10.1097/BRS.0000000000001810

[5] Mo X , Shen J , Jiang W , *et al*. Percutaneous endoscopic lumbar diskectomy for axillar herniation at L5–S1 via the transforaminal approach versus the Interlaminar approach: a prospective clinical trial. World Neurosurg, 2019, 125: e508–e514. 30710722 10.1016/j.wneu.2019.01.114

[6] Chen J , Jing X , Li C , Jiang Y , Cheng S , Ma J . Percutaneous endoscopic lumbar discectomy for L5S1 lumbar disc herniation using a Transforaminal approach versus an Interlaminar approach: a systematic review and meta‐analysis. World Neurosurg, 2018, 116: 412–420. 29783008 10.1016/j.wneu.2018.05.075

[7] Choi KC , Kim JS , Ryu KS , Kang BU , Ahn Y , Lee SH . Percutaneous endoscopic lumbar discectomy for L5–S1 disc herniation: transforaminal versus interlaminar approach. Pain Physician, 2013, 16: 547–556. 24284840

[8] Schultz A , Andersson G , Ortengren R , Haderspeck K , Nachemson A . Loads on the lumbar spine. Validation of a biomechanical analysis by measurements of intradiscal pressures and myoelectric signals. J Bone Joint Surg Am, 1982, 64: 713–720. 7085696

[9] Yin J , Jiang Y , Nong L . Transforaminal approach versus interlaminar approach: a meta‐analysis of operative complication of percutaneous endoscopic lumbar discectomy. Medicine, 2020, 99: e20709. 32569205 10.1097/MD.0000000000020709 PMC7310843

[10] Cheng L , Cai H , Liu Z , Yu Y , Li W , Li Q . Modified full‐endoscopic Interlaminar discectomy via an inferior endplate approach for lumbar disc herniation: retrospective 3‐year results from 321 patients. World Neurosurg, 2020, 141: e537–e544. 32492545 10.1016/j.wneu.2020.05.234

[11] Choi G , Lee SH , Raiturker PP , Lee S , Chae YS . Percutaneous endoscopic interlaminar discectomy for intracanalicular disc herniations at L5–S1 using a rigid working channel endoscope. Neurosurgery, 2006, 58: ONS59–ONS68. 16479630 10.1227/01.neu.0000192713.95921.4a

[12] Sencer A , Yorukoglu AG , Akcakaya MO , *et al*. Fully endoscopic interlaminar and transforaminal lumbar discectomy: short‐term clinical results of 163 surgically treated patients. World Neurosurg, 2014, 82: 884–890. 24907438 10.1016/j.wneu.2014.05.032

[13] Xin Z , Huang P , Zheng G , Liao W , Zhang X , Wang Y . Using a percutaneous spinal endoscopy unilateral posterior interlaminar approach to perform bilateral decompression for patients with lumbar lateral recess stenosis. Asian J Surg, 2020, 43: 593–602. 31594687 10.1016/j.asjsur.2019.08.010

[14] Li ZZ , Hou SX , Shang WL , Song KR , Zhao HL . The strategy and early clinical outcome of full‐endoscopic L5/S1 discectomy through interlaminar approach. Clin Neurol Neurosurg, 2015, 133: 40–45. 25837573 10.1016/j.clineuro.2015.03.003

